# Update Notice: Computational Analysis of Plasma Lipidomics from Mice Fed Standard Chow and Ketogenic Diet

**DOI:** 10.21769/BioProtoc.4884

**Published:** 2023-10-20

**Authors:** Amy L. Seufert, James W. Hickman, Jaewoo Choi, Brooke A. Napier

**Affiliations:** 1 Department of Biology and Center for Life in Extreme Environments, Portland State University, Portland, OR, USA; 2 Linus Pauling Institute, Oregon State University, Corvallis, OR, USA

After official publication in Bio-protocol (https://bio-protocol.org/e4819), we would like to add the following source of funding to the Acknowledgments section of the article: “The QTOF mass spectrometer was funded by an instrument grant from the NIH: ABSciex Triple ToF 5600 NIH #1S10RR027878-01.”

